# Characterization of Soybean Protein Isolate-Food Polyphenol Interaction via Virtual Screening and Experimental Studies

**DOI:** 10.3390/foods10112813

**Published:** 2021-11-16

**Authors:** Le Ao, Panhang Liu, Annan Wu, Jing Zhao, Xiaosong Hu

**Affiliations:** 1 College of Food Science and Nutritional Engineering, China Agricultural University, Beijing 100083, China; a.kamio@163.com (L.A.); panhangliu@163.com (P.L.); s20213061005@cau.edu.cn (A.W.); huxiaos@263.net (X.H.); 2 China National Engineering Research Center for Fruit and Vegetable Processing, Beijing 100083, China; 3 Key Laboratory of Fruit and Vegetable Processing, Ministry of Agriculture, Beijing 100083, China; 4 China Academy of Machinery Science and Technology Group Co., Ltd., Beijing 100083, China

**Keywords:** soybean protein isolate, polyphenol, molecular docking, fluorescence

## Abstract

(1) Background: Protein–polyphenol interactions have been widely studied regarding their influence on the properties of both protein and the ligands. As an important protein material in the food industry, soybean protein isolate (SPI) experiences interesting changes through polyphenols binding. (2) Methods: In this study, a molecular docking and virtual screening method was established to evaluate the SPI–polyphenol interaction. A compound library composed of 33 commonly found food source polyphenols was used in virtual screening. The binding capacity of top-ranking polyphenols (rutin, procyanidin, cyanidin chloride, quercetin) was validated and compared by fluorescence assays. (3) Results: Four out of five top-ranking polyphenols in virtual screening were flavonoids, while phenolic acids exhibit low binding capacity. Hydrogen bonding and hydrophobic interactions were found to be dominant interactions involved in soybean protein–polyphenol binding. Cyanidin chloride exhibited the highest apparent binding constant (Ka), which was followed by quercetin, procyanidin, and rutin. Unlike others, procyanidin addition perturbed a red shift of SPI fluorescence, indicating a slight conformational change of SPI. (4) Conclusions: These results suggest that the pattern of SPI–polyphenol interaction is highly dependent on the detailed structure of polyphenols, which have important implications in uncovering the binding mechanism of SPI–polyphenol interaction.

## 1. Introduction

Protein–polyphenol interaction has been attracting more and more attention due to its beneficial influence to the properties of both the protein and polyphenols. Soybean protein isolate (SPI) is one of the most important protein products prepared from soybean. With the development of emerging plant-based foods, there are considerable interest in expanding the utilization of plant proteins because of their potential benefits in terms of sustainability, health, and ethical issues. However, some natural properties of SPI cannot meet the commercial needs, limiting the application of SPI in food processing. Recently, many studies indicate that the solubility, emulsifiability, thermal stability, and functional properties of SPI are altered when SPI is complexed with small molecules, especially polyphenol compounds [1,2]. EGCG binds SPI and enhances the foaming and emulsifying activity of SPI [3]. The forming of a rutin–SPI complex significantly increased the foaming capacity of SPI [4]. The underlying mechanisms include the exposure of hydrophobic amino acid residues, the change of protein secondary structure, the bridging of protein–solvent interactions, etc. In another aspect, protein–polyphenol binding was also reported to modify the property of polyphenol itself, stabilizing the compound and increasing its bioavailability and bioactive functions such as antioxidant activity [5]. 

More and more polyphenol compounds are being tested for their protein binding capacities, while rare research had been carried out on the comparison of the behavior of different polyphenols. Dietary polyphenols exhibit many functional activities, with distinct structures varying with different categories and sources [6,7,8,9,10]. It is mainly composed of five categories, including flavonoids, phenolic acids, stilbenes, lignans and their derivatives, among which flavonoids are the most abundant [11]. Flavonoids are characterized by a unique C3–C6–C3 skeleton and further divided into six main subclasses including catechins, flavanols, flavones, flavanones, isoflavones, and anthocyanidins. Phenolic acids are featured by carboxylic acid groups, and stilbenes contain two benzene rings joined by a molecule of ethylene [12]. Lignans are phenolic dimers and oligomers possessing a 2,3-dibenzylbutane structure [13]. Distinct structures endow polyphenol compounds with miscellaneous physiochemical properties, likely affecting their protein-binding behavior. Thus, the characterization and comparison of protein–polyphenol interaction is important to understand how SPI interacts with different polyphenols, which has implications on the utilization of SPI–polyphenol complex in food industry. 

In this study, 33 polyphenol compounds were selected from common food sources, including 25 flavonoids, 6 phenolic acids, 1 lignan, and 1 stilbenes, and compared regarding their SPI-binding capacity. The SPI–polyphenol interactions were investigated by molecular docking, virtual screening, and fluorescence quenching assays. Our work offers references for uncovering the SPI–polyphenol binding mechanisms and the utilization of polyphenols in SPI modification.

## 2. Materials and Methods

### 2.1. Materials

Soy protein isolate (90.0% protein) used in this study was purchased from Shandong Biological Products Co., Ltd. (Linyi, China). Rutin, lignan, procyanidin, cyanidin chloride, and quercetin (purity > 90%) were purchased from Solarbio Life Science Ltd. (Beijing, China). All other chemicals and regents were of analytical grade. Syringe filters were produced from Sigma–Aldrich (St. Louis, MO, USA).

### 2.2. Binding Pockets Identification by Schrödinger Suites

Schrödinger suites [14,15] (Schrödinger Release 2021-3: SiteMap, Schrödinger, LLC, New York, NY, USA, 2021) was used to predict the binding pockets on soybean 11S glycinin. The crystal structure of 11S glycinin (PDB ID: 1OD5) was prepared using the Protein Preparation Wizard in Maestro. Hydrogens were added, and the bond orders and formal charges were assigned. The hydrogen bond network of the protein was optimized, including the reorientation of hydroxyl and thiol groups and the prediction of the protonation states of aspartic acid, glutamic acid, and histidine. A brief minimization was carried out after the H-bond network optimization. The SiteMap module of Schrödinger was used to identify potential binding sites following a standard protocol.

### 2.3. EGCG Docking and Pocket Selection

As a known SPI binder, epigallocatechin gallate (EGCG) was used as a model compound and docked into the pockets predicted by Schrödinger Sitemap. Schrödinger Glide was used in molecular docking operations. The grid files were generated by the Receptor Grid Generation module after importing the protein structure prepared. The docking of EGCG into pockets was evaluated by the ligand docking function. The docking scores were used to select the most appropriate binding pocket. 

### 2.4. Virtual Screening of Food Source Polyphenols

A virtual screening was carried out to evaluate the binding capacity of 33 commonly used polyphenols from different food sources (including rutin, procyanidin, quercetin, resveratrol, gallic acid, etc., see Supplemental Table S1) to SPI. The screening was performed using Schrödinger Glide based on the grid files generated in Section 2.3. The selected polyphenols were docked into the optimal pocket and evaluated by the ligand docking function. The molecular conformation change deviation was investigated by calculating the root mean square deviation (RMSD) of each atom. RMSD values of less than 2 were considered as successful in reproducing the protein–ligand interaction.

### 2.5. Validation of SPI–Polyphenol Binding by Fluorescence Assays

The intrinsic fluorescence spectra were measured using an F-7000 spectrofluorometer (HITACHI, Tokyo, Japan) at 25 °C with a cell of 1 cm path length. The fluorescence was measured by exciting the protein at 280 nm and recording its emission spectra in the wavelength range of 310–450 nm. The final protein concentration was kept at 0.135 mg/mL. A series of aliquots of polyphenol solution (0.1 mM) were sequentially added to 4 mL of SPI solution (0.135 mg/mL). 

The fluorescence data were calculated using the Stern–Volmer equation:

*F*_0_/*F* = 1 + *K_sv_*[*Q*]. (1)

In this equation, *F* and *F*_0_ are the fluorescence intensity of the protein solution with polyphenols and without, respectively, *K_sv_* is the quenching constant, and [*Q*] is the concentration of polyphenols. 

Binding parameters can be estimated using the quenching data by equation:

*log*[(*F*_0_ − *F*)/*F*] = *logK_a_* + *nlog*[*Q*]. (2)

In this equation, *K_a_* is the apparent binding constant, and *n* is the number of binding sites.

### 2.6. Statistical Analysis

The statistical analysis software GraphPad (San Diego, CA, USA) was used to analyze the data. A comparison of the means was ascertained by Duncan’s test at a 5% level of significance using one-way analysis of variance (ANOVA).

## 3. Results and Discussion

### 3.1. Binding-Pocket Identification and EGCG Docking

**Figure 1:**
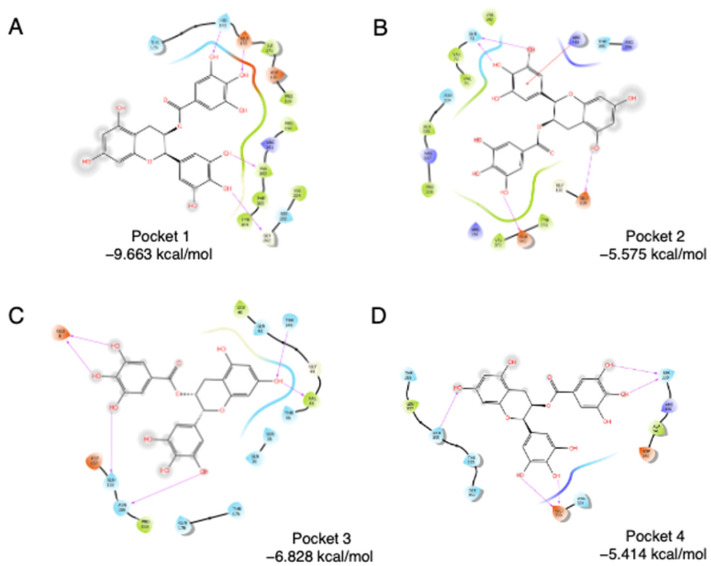
Docking of EGCG into four pockets on soybean 11S globulin. (**A**) Pocket 1, (**B**) Pocket 2, (**C**) Pocket3, (**D**) Pocket4.

SPI is mainly composed of glycinin (11S), β-conglycinin (7S), and lipophilic proteins [16,17]. 11S glycinin has been used to construct hollow microcapsule as a delivery system for bioactive compounds [18]. Using a Schrödinger Sitemap, four binding pockets were obtained on soybean 11S glycinin. To evaluate the accessibility of four pockets, molecular docking was carried out using epigallocatechin gallate (EGCG), a known SPI binder [3,19], as the ligand. As shown in Figure 1, EGCG was docked into the four pockets individually. Pocket 1 has the lowest binding energy, which is −9.663 kcal/mol, followed by Pocket 3 (−6.828 kcal/mol), Pocket 2 (−5.575 kcal/mol), and Pocket 4 (−5.414 kcal/mol). For Pocket 1, direct interactions were found between the phenol groups of EGCG, especially ring B and ring D, and amino acids of 11S. Glu172, His173, Val162, and Gly202 are highly involved in the interaction. Two hydroxyl groups on the B ring interact with Val162 and Gly202, separately. Two hydroxyl groups on the D ring interact with Glu172 and His173, separately. Thus, pocket 1 was chosen as the binding region in the following assays.

### 3.2. Virtual Screening of Food Polyphenols against Soybean Protein

**Figure 2:**
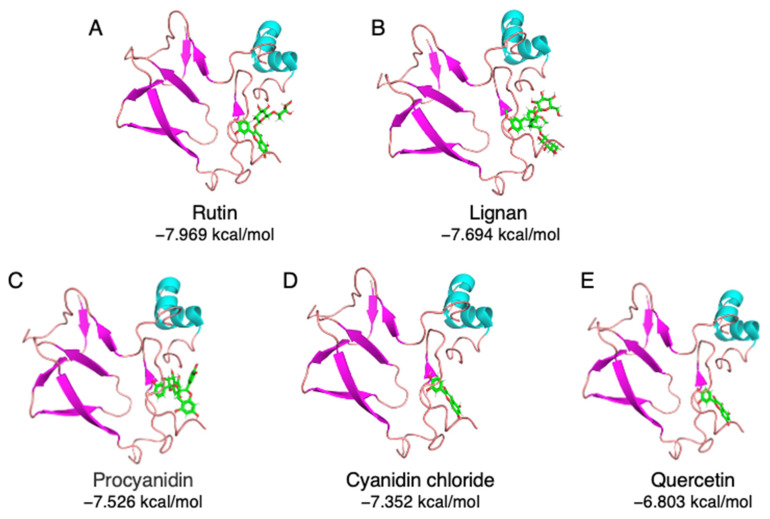
The stereo view of top-ranking polyphenols complexed with soybean 11S globulin. (**A**) Rutin, (**B**) lignan, (**C**) procyanidin, (**D**) cyanidin chloride, and (**E**) quercetin.

**Figure 3:**
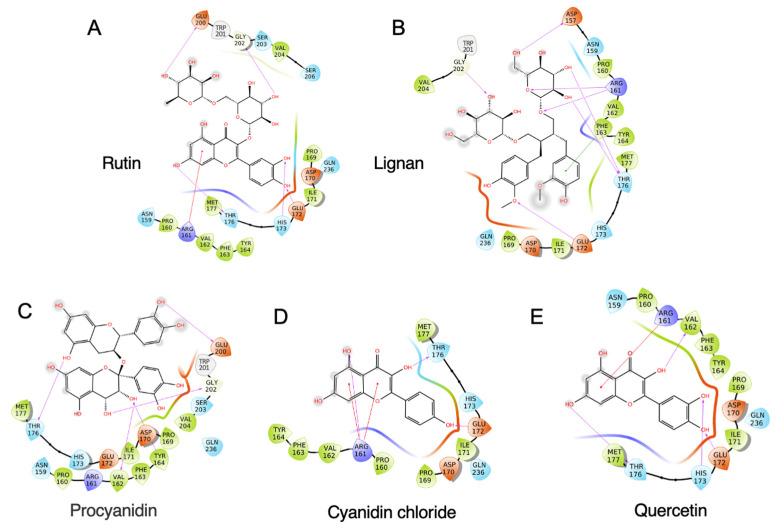
Interaction diagram of top-ranking polyphenols complexed with soybean 11S globulin. (**A**) Rutin, (**B**) lignan, (**C**) procyanidin, (**D**) cyanidin chloride, and (**E**) quercetin.

A total of 33 commonly used polyphenols from different food sources were selected, including rutin, procyanidin, quercetin, resveratrol, gallic acid, etc. (Supplemental Table S1). A virtual screening was carried out to evaluate the binding capacity of different food polyphenols. As shown in Supplemental Table S1, rutin exhibited the lowest binding energy, which is −7.969 kcal/mol, followed by lignan (−7.694 kcal/mol) and procyanidin (−7.526 kcal/mol). The stereo view of the selected 11S–polyphenol complex is shown in Figure 2. Detailed interactions are shown in Figure 3. Both hydrogen bonding and hydrophobic interaction are likely involved in the rutin–11S complex [3,20]. A substantial amount of hydrogen bondings could form between 11S amino acid side-chain protons and ring oxygens of rutin (Figure 3A), which likely contribute to its binding to SPI. The major amino acids involved in rutin binding include Glu172, His 173, Thr176, Arg 161, Glu200, and Gly202. For lignan, amino acids including Asp157, Arg161, Phe163, Thr176, Glu172, and Gly202 dominate the binding (Figure 3B). For procyanidin (Figure 3C), Val162, Tyr164, Thr176, Glu200, and Gly202 are highly involved. Less interactions were found for cyanidin chloride (Figure 3D) and quercetin (Figure 3E), with cyanidin chloride binding to Arg161, Glu172, Thr176, and quercetin binding to Arg161, Val162, Glu172, His173, and Met177.

### 3.3. Validation of Polyphenol–SPI Interaction by Fluorescence Quenching

Fluorescence quenching assay has been widely used to characterize protein–ligand interactions [2,21]. Aromatic amino acids (mainly tryptophan and tyrosine) in protein can be excited by a laser wavelength around 280 and emitted a strong fluorescence signal, a.k.a. the intrinsic protein fluorescence [22]. Quencher molecules decrease the intrinsic fluorescence of protein in certain patterns. Polyphenol compounds have been reported to be fluorescence quenchers, and thus, the protein–polyphenol interaction can be detected by fluorescence assay [23]. In this study, the five top-ranking compounds from virtual screening, rutin, lignan, procyanidin, cyanidin chloride, and quercetin, were applied in fluorescence quenching assay to validate binding).

**Figure 4:**
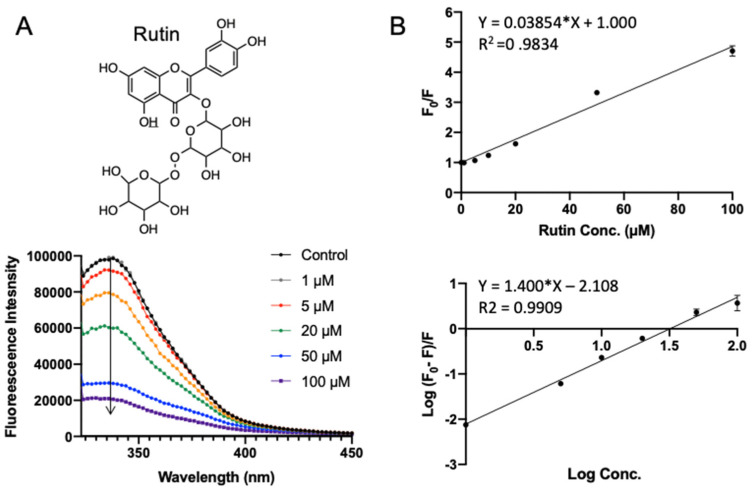
Fluorescence assay of SPI–rutin interaction. (**A**) The fluorescence emission profiles of SPI in the presence of different concentrations of rutin. The inset corresponds to the molecular structure of rutin. (**B**) The Stern–Volmer plot and binding parameter estimation plot.

Rutin (quercetin-3-O-rutinoside) is a glycoside flavonoid abundantly found in many plants, such as buckwheat, apples, and tea [24]. It is reported to have antioxidant, antiinflammation, anti-tumor, and neuroprotective activities [25,26,27,28]. Rutin exhibited the lowest binding energy (−7.969 kcal/mol) in molecular docking. Figure 4 shows the fluorescence emission spectra of SPI with the addition of rutin. With the increase in rutin concentration (from 1 to 100 μΜ), the fluorescence intensity of SPI decreased progressively, confirming the interaction between rutin and SPI. As shown in Figure 4B (top), *F*_0_*/F* was fitted well against rutin concentration by the Stern–Volmer equation (Equation (1)). A linear Stern–Volmer plot was observed, which means that only one type of quenching mechanism occurs (dynamic or static). The quenching constant *K_sv_* was fitted to be (0.039 ± 0.0009) × 10^6^ L·mol^−1^ (Table 1). Binding parameters, the apparent binding constant *K_a_*, and the average number of binding sites *n* were obtained by fitting experimental data to Equation (2). It turns out that the rutin–SPI interaction exhibited a *K_a_* of (0.007 ± 0.001) × 10^6^ L·mol^−1^ and an average number of binding sites as 1.40 ± 0.034. 

**Figure 5:**
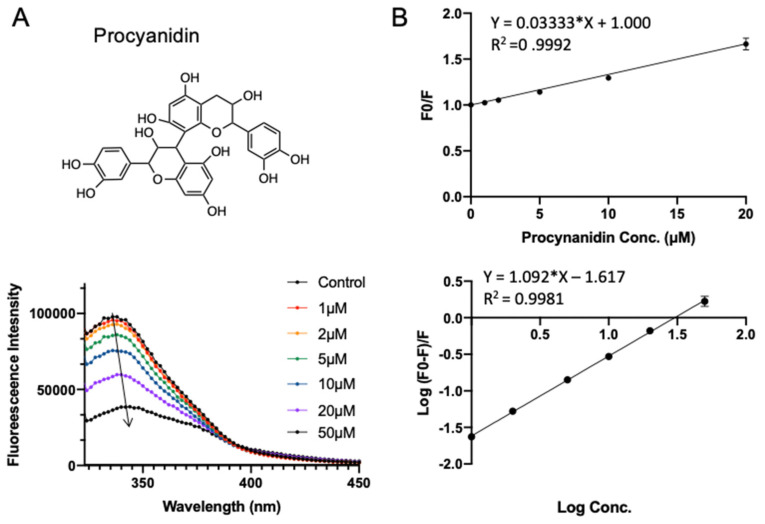
Fluorescence assay of SPI–procyanidin interaction. (**A**) The fluorescence emission profiles of SPI in the presence of different concentrations of procyanidin. The inset corresponds to the molecular structure of procyanidin. (**B**) The Stern–Volmer plot and binding parameter estimation plot.

Procyanidin is the most common subgroup of proanthocyanidins, which is commonly found in fruits (especially berries), some cereals, and root vegetables (onions cabbages, and radishes). It is a dimer or oligomer composed of epicatechin units and their galloyl derivatives [29,30]. The procyanidin used in this study is a B-type dimer (B2) construct. Procyanidin B2 has been reported to have beneficial effects on cardiovascular diseases and aging [31,32], which was claimed to be a potent inhibitor of COVID-19 protease recently [33]. It has a docking energy of −7.526 kcal/mol in virtual screening. Similar to rutin, adding procyanidin induced the decrease in intrinsic fluorescence intensity of SPI. Notably, a red shift (from 347 to 349 nm) is observed with increasing procyanidin concentration (from 1 to 50 μΜ; see Figure 5A), suggesting that the fluorophores of SPI are located in a more hydrophilic environment in the presence of procyanidin [22], which indicates that procyanidin may induce slight conformational changes of SPI. As shown in Figure 5B and Table 1, the quenching constant *K_sv_* for procyanidin was fitted to be (0.033 ± 0.0003) × 10^6^ L·mol^−1^. The apparent binding constant *K_a_* and the average number of binding sites *n* were obtained to be (0.024 ± 0.002) × 10^6^ L·mol^−1^ and 1.09 ± 0.011, respectively.

**Figure 6:**
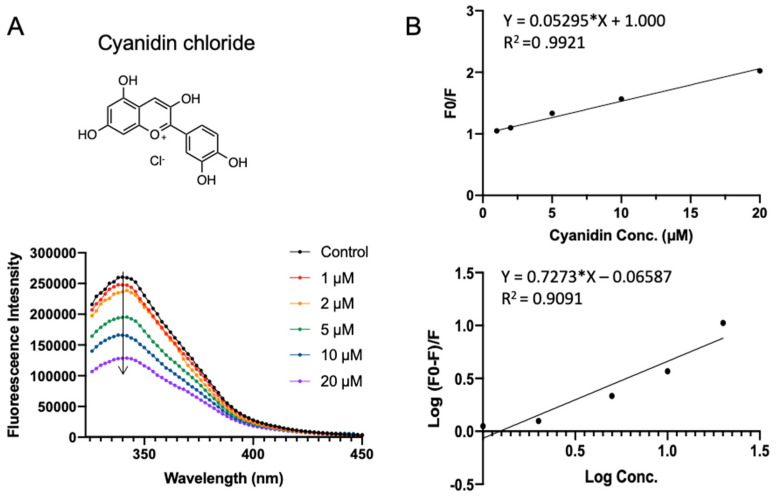
Fluorescence assay of SPI–cyanidin chloride interaction. (**A**) The fluorescence emission profiles of SPI in the presence of different concentrations of cyanidin chloride. The inset corresponds to the molecular structure of cyanidin chloride. (**B**) The Stern–Volmer plot and binding parameter estimation plot.

Figure 6 shows the SPI fluorescence quenching by cyanidin chloride, which has a docking energy of −7.401 kcal/mol in virtual screening. Cyanidin chloride is a depolymerization product of procyanidin or naturally occurring compound that is found in berries, flowers, and vegetables. Compared to procyanidin, cyanidin chloride did not perturb the red shift of SPI fluorescence, indicating the existence of a different binding mode. A similar quenching constant *K_sv_* of (0.053 ± 0.0010) × 10^6^ L·mol^−1^ was obtained (Table 1). Cyanidin has the highest apparent binding constant *K_a_* of (0.861 ± 0.039) × 10^6^ L·mol^−1^ and the average number of binding sites *n* fitted to be 0.73 ± 0.064.

**Figure 7:**
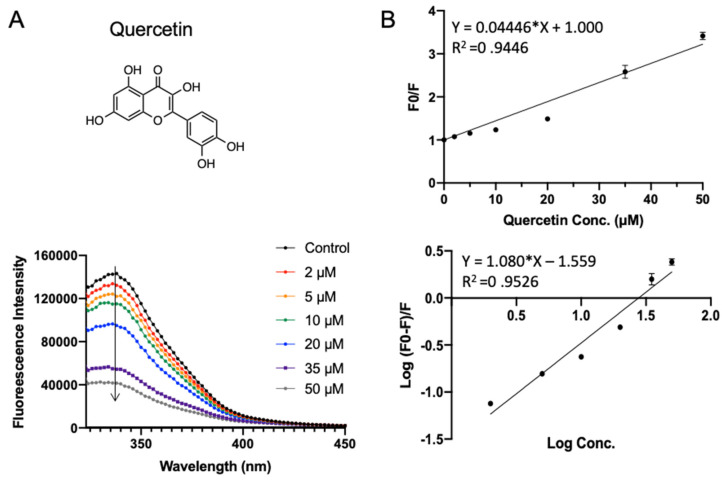
Fluorescence assay of SPI–quercetin interaction. (**A**) The fluorescence emission profiles of SPI in the presence of different concentrations of quercetin. The inset corresponds to the molecular structure of quercetin. (**B**) The Stern–Volmer plot and binding parameter estimation plot.

Quercetin widely exists in our daily diet and has a wide range of biological functions including anti-inflammatory, antiviral, and anti-carcinogenic activities, as well as attenuating lipid peroxidation and platelet aggregation [34]. In addition to natural occurrence, it can also be released in the gastrointestinal tract from hydrolysis after oral administration of rutin. As shown in Figure 7A, quercetin quenched the intrinsic fluorescent of SPI in a concentration-dependent manner. The binding parameters fitted in Figure 7B are shown in Table 1. Quercetin exhibited a similar *K_sv_* of (0.044 ± 0.0017) × 10^6^ L·mol^−1^ as the other compounds. The apparent binding constant *K_a_* and the average number of binding sites *n* were fitted to be (0.028 ± 0.003) × 10^6^ L·mol^−1^ and 1.08 ± 0.060, respectively. 

## 4. Conclusions

Numerous polyphenol compounds had been reported regarding their protein binding capacities, while rare research had been carried out on the comparison of the behavior of different polyphenols. In this study, 33 dietary polyphenol compounds including 25 flavonoids, 6 phenolic acids, 1 lignan, and 1 stilbene were analyzed and compared regarding their SPI binding capacity. Molecular docking, virtual screening, and fluorescence quenching assays were performed. Interestingly, four out of five top-ranking polyphenols in virtual screening were flavonoids, while phenolic acids exhibit relatively low binding capacity. It suggests that the SPI–polyphenol interaction is highly dependent on the variety of polyphenols. In vitro SPI binding of top-ranking polyphenols (rutin, procyanidin, cyanidin chloride, quercetin) was tested and compared by fluorescence quenching assays. Cyanidin chloride exhibited the highest apparent binding constant *K_a_*, while procyanidin perturbed a red shift of SPI fluorescence, indicating a slight conformational change of SPI. These results indicate that polyphenols with a different structure may exhibit distinct protein binding patterns. Our work provides a combined strategy of molecular docking, virtual screening, and in vitro validation assays in charactering the SPI–polyphenol interactions, which has important implications in uncovering the SPI–polyphenol binding mechanism on a molecular level and offers a fundamental basis for the utilization of the SPI–polyphenol complex in food systems. 

## Figures and Tables

**Table 1:** Binding parameters of selected SPI–polyphenol interactions.

Number	*K_SV_* (×10^6^ L·mol^−1^)	*n*	*K_a_* (×10^6^ L·mol^−1^)
rutin	0.039 ± 0.0009 ^c^	1.40 ± 0.034 ^a^	0.007 ± 0.001 ^d^
procyanidin	0.033 ± 0.0003 ^d^	1.09 ± 0.011 ^b^	0.024 ± 0.002 ^c^
cyanidin chloride	0.053 ± 0.0010 ^a^	0.73 ± 0.064 ^c^	0.861 ± 0.039 ^a^
quercetin	0.044 ± 0.0017 ^b^	1.08 ± 0.060 ^b^	0.028 ± 0.003 ^b^

^a,b,c,d^ Values with different superscript letters in a column are significantly different (*p* < 0.05).

## Data Availability

Not applicable.

## Supplementary Materials

The following are available online at https://www.mdpi.com/article/10.3390/foods10112813/s1, Table S1: The docking scores of 33 commonly found food polyphenols.
