# Supplementary material for: Characterization of Soybean Protein Isolate-Food Polyphenol Interaction via Virtual Screening and Experimental Studies
Source: Foods. 2021 Nov 16;10(11):2813. doi: 10.3390/foods10112813 (PMC8625844; doi:10.3390/foods10112813)
Supplement: Supplementary file 1 [file foods-10-02813-s001.zip › foods-1440129-supplementary.pdf]

Table S1. The docking scores of 33 commonly found food polyphenols

Number	CAS	Category	Dietary Polyphenol	Docking Score （kcal/mol）
1	153-18-4	flavonoid	rutin	-7.969
2	148244-82-0	lignan	seco-isolariciresinol diglucoside	-7.694
3	29106-49-8	flavonoid	Proanthocyanin B2	-7.526
4	528-58-5	flavonoid	cyanidin chloride	-7.401
5	117-39-5	flavonoid	quercetin	-7.352
6	134-01-0	flavonoid	peonidin chloride	-7.297
7	18829-70-4	flavonoid	(-)-catechin hydrate	-6.803
8	490-46-0	flavonoid	L-epicatechin	-6.722
9	528-48-3	flavonoid	fisetin	-6.654
10	491-80-5	flavonoid	5,7-dihydrox-4'-methoxyisoflavone	-6.652
11	60-82-2	flavonoid	phloretin	-6.648
12	40957-83-3	flavonoid	glycitein	-6.584
13	529-44-2	flavonoid	myricetin	-6.536
14	970-74-1	flavonoid	(-)-epigallocatechin	-6.511
15	520-18-3	flavonoid	kaempferol	-6.483
16	989-51-5	flavonoid	(-)-epigallocatechin gallate	-6.474
17	490-79-9	phenolic acid	2,5-dihydroxybenzoic acid	-6.458
18	520-36-5	flavonoid	apigenin	-6.444
19	501-36-0	stilbene	resveratrol	-6.428
20	491-70-3	flavonoid	luteolin	-6.424
21	60-81-1	flavonoid	phlorizin	-6.327
22	552-58-9	flavonoid	eriodictyol	-6.275
23	1257-08-5	flavonoid	(-)-epicatechin gallate	-6.201
24	446-72-0	flavonoid	genistein	-6.166
25	99-50-3	phenolic acid	3,4-dihydroxybenzoic acid	-5.905
26	491-67-8	flavonoid	baicalein	-5.857
27	486-66-8	flavonoid	daidzein	-5.846
28	149-91-7	phenolic acid	gallic acid	-5.646
29	458-37-7	flavonoid	curcumin	-5.32
30	1135-24-6	phenolic acid	ferulic acid	-5.289
31	327-97-9	phenolic acid	chlorogenic acid	-5.164
32	331-39-5	phenolic acid	caffeic acid	-4.955
33	10236-47-2	flavonoid	naringin	-4.757
